# The Effect of Comprehensive Use of PDCA and FMEA Management Tools on the Work Efficiency, Teamwork, and Self-Identity of Medical Staff: A Cohort Study with Zhongda Hospital in China as an Example

**DOI:** 10.1155/2022/5286062

**Published:** 2022-05-23

**Authors:** Hui Chen, Zhao Tao, Chen Zhou, Su Zhao, Yanan Xing, Min Lu

**Affiliations:** ^1^Medical Division, Zhongda Hospital, Southeast University, Nanjing 210009, China; ^2^Medical Insurance Office, Zhongda Hospital, Southeast University, Nanjing 210009, China

## Abstract

**Objective:**

Taking Zhongda Hospital in China as an example, this study discusses the impact of comprehensive use of PDCA and FMEA management tools on the work efficiency, teamwork, and self-identity of medical staff.

**Methods:**

Two hundred medical staff in our hospital from January 2020 to December 2021 were selected as the research subjects, and the 200 medical staff were divided into a control group and a research group by the digital table method, with 100 cases in each group. The medical staff in the control group implemented conventional system management methods, while the research group comprehensively used PDCA and FMEA management tools based on implementing conventional system management. The differences in work efficiency, teamwork, and self-identity of medical staff were compared.

**Results:**

Before the study, there exhibited no significant difference in the work efficiency, teamwork, and self-identity scores of medical staff (*P* > 0.05). After an intervention, the work efficiency, teamwork, and self-identity scores of medical staff in the study group were higher than those in the control group (*P* < 0.05). After the intervention, the management quality score of the research group was higher than that of the control group (*P* < 0.05); after the intervention, the medical staff in the research group had lower work efficiency, insufficient professional ability, and insufficient management system cognitive behaviours than that in the control group (*P* < 0.05).

**Conclusion:**

The comprehensive use of PDCA and FMEA management tools in internal hospital management can remarkably enhance the work efficiency, teamwork, and self-identity of medical staff.

## 1. Introduction

The internal management system of a hospital, that is the hospital rules and regulations, refers to the general term for the normative documents issued by the hospital to adjust various management relations such as hospital medicine, teaching, research, prevention, management, and operation, which are generally binding in the whole hospital [1]. Standardized management is actually to digitize and quantify every aspect of the management work through various management means and methods to enhance the organization's execution efficiency and execution strength. At present, the main mode of hospital management at all levels is hierarchical management. Few hospitals can apply a systematic and lasting good management mode. Many hospitals remain reactive to various examinations and assessments [2]. Therefore, in the quality construction of hospitals in my country, it is of great practical significance to update management concepts on time, innovate management methods, and actively explore a hospital management model that is suitable for its own development with good operating mechanism [3]. A scientific and fair hospital quality evaluation system is established: scientific, standardized, rigorous, and practical evaluation standards are formulated, which are directly related to the effect of medical quality management. While implementing the advanced quality management model, secondary assessment and evaluation standards are established so that all employees can participate in the process of quality management and achieve the effect of full participation [4]. The first is the department quality assessment standard system: in accordance with the relevant health laws and regulations, combined with the actual situation of the hospital, various functional departments are organized to formulate and improve a series of scientific, reasonable, and operable medical rules and regulations, such as surgical classification system and abnormal medical treatment information reporting system, so that each work has a legal basis, to standardize the daily work of medical care. The second is the individual quality evaluation system: two different groups evaluate individuals according to the same standard, avoid one-time defects, and achieve scientific and rational evaluation [5].

Dr. Deming, an American quality management expert, put forward the concept of plan, do, check, and act (PDCA) cycle in the 1950s. At the same time, it is widely used in the quality management of various industries [6]. It can make the cycle system of various industries more procedural, standardized, and scientific [7]. The PDCA cycle management tool, as a scientific procedure followed by total quality management, refers to the application of the four-stage management model of plan, execution (do), inspection (check), and processing (action) to any workflow, forming A closed-loop quality management model in PDCA sequence [8, 9]. The PDCA cycle management theory is applied to the internal system management of the hospital, and it has relatively significant management advantages, including the following: (1) the PDCA cycle management theory improves the staff and other management subjects' cognition of internal system management and then stimulates the management subjects' sense of responsibility and work enthusiasm, to achieve the purpose of optimizing the hospital's internal management system. (2) This management theory aims to maintain the normal operation of the hospital and enhance the service quality and emphasizes the importance of each staff member. (3) When the internal system manager loses absolute control and personal authority, it is to give each staff member the corresponding management power, fully support the work of each staff member, and enhance harmonious communication between staff members. Professional guidance and training provide important prerequisites for staff to complete their work with high quality. (4) The work enthusiasm and sense of responsibility of the internal management staff are effectively stimulated, and the quality of internal management work is improved by understanding the work schedule and plan and clarifying the work goals. (5) The quality of internal management work has been continuously improved, and the smooth development of internal management work has been planned and progressed [10]. Failure mode and effect analysis (FMEA) is a team-based, systematic, and forward-looking analysis method used to identify how and why a process fails in a design and provide recommendations and develop improvement measures for improving the failure. It is a continuous quality improvement process with quantifiable and forward-looking advantages, which can systematically identify failure modes in medical services, especially process management, and attach importance to promoting medical quality and safety management. This tool first originated in the aviation field in the United States and later received attention and was widely adopted in some medical fields [11, 12]. After introducing FMEA risk management tools in my country, its application in hospital management has gradually increased in recent years, covering multiple hospital management fields such as nursing, infection, and pharmaceutical affairs. Based on this, this study explores the impact of the comprehensive use of PDCA and failure mode and effect analysis (FMEA) management tools on the work efficiency, teamwork, and self-identity of medical staff. The results of the study are reported as follows.

## 2. Cases and Methods

### 2.1. General Information

Two hundred medical staff in our hospital from January 2020 to December 2021 were prospectively enrolled as the research subjects, and the 200 medical staff were assigned into a control group and a research group by the digital table method, with 100 cases in each group. The medical staff in the control group implemented conventional system management, while the research group comprehensively used PDCA and FMEA management based on implementing conventional system management. Among them, the age of the research group was 27–52 years; the age of the control group was 28–51 years. There exhibited no significant difference in general data such as gender and age of subjects (*P* > 0.05), and they are comparable, as indicated in Table 1. All patients were aware of the study protocol and signed the consent form, which was permitted by the ethics committee of our hospital.

  Inclusion criteria are as follows: (1) college degree or above and (2) voluntary participation in this research and signed informed consent.
  Exclusion criteria are as follows: (1) those who go out for further study, those who are on sick leave, and those who are on maternity leave; (2) those who are newly recruited, transferred to departments or temporarily assigned to the department during the study period.

### 2.2. Treatment Methods

The medical staff in the control group implemented conventional system management methods. The research group comprehensively uses PDCA and FMEA management tools based on implementing conventional system management: establishes an FMEA team, trains and educates team members on FMEA-related knowledge, conducts discussions on the hospital's internal system management, and analyzes the failure modes of each process. They are listed one by one, all possible failures and invalid problems are found, and the reasons for them are analyzed; the risk priority index is calculated: that is, the product of the risk severity (severity, S), the risk occurrence rate (occurrence, O), and the risk detection degree (detection, D), which is adopted to measure the possible defects. The larger the value between 1 and 1000, the more serious the potential problem and the higher the risk. Afterwards, the PDCA cycle method will be adopted to implement standardized system management methods [8]: (1) formulate plans: the quality management department will comprehensively sort out and revise the rules and regulations of the whole hospital in combination with the grade review rules and establish the hospital system management committee, and the training department will lead the various functional departments in the whole hospital. System training is carried out. (2) Implementation stage: It includes drafting of the normative system, normative format. Only a standardized system can ensure its authority, effectively enhance the hospital management efficiency, ensure the normal operation of the hospital's work, and promote the high-quality development of the hospital. Taking the grading review of general tertiary hospitals as an opportunity, combined with the relevant requirements of performance appraisal of public hospitals, the hospital management system is comprehensively sorted. Led by the hospital quality management department, each functional department revises the hospital-level management system and compiles it into a book. Clinical and medical technology departments revise the relevant management systems of their respective departments and form department quality management manuals. Before drafting the system, in-depth investigations are conducted, relevant materials are collected, and the opinions of relevant parties in various forms are listened, such as written comments, online comments, symposiums, demonstration meetings, and discussions at the workers' congress. The drafting department shall solicit the opinions of employees for systems involving the basic rights or vital interests of employees. If the content involves the functions and powers of multiple functional departments, it is jointly drafted by two or more functional departments. After the drafting department completes the typesetting of the manuscript for review according to the system template, it initiates an application in the OA system. The format and font specifications of all systems are unified. The hospital-level system is the standardized management document of the hospital, and each volume is numbered. The upper walls of all systems also formulate relevant regulations and designate responsible departments. Standardized system review and unified export: the hospital establishes a system committee, and each functional department drafts the relevant system and applies it for the hospital OA. All hospital-level systems need to be reviewed and approved by 2/3 of the system committee before entering the next stage of OA. The audit of the members of the system committee is mainly carried out from the aspects of legitimacy, rationality, and coordination. The system that has passed the review will be discussed at the dean's office meeting, and the hospital office will publish the system that has been discussed and passed. The department-level management system is drafted according to its responsibilities and authority, discussed and approved by the department's quality management team, and submitted to the competent functional department for review. The quality management department is the only outlet of the system, which is responsible for reviewing the text and text of the management system and classifying, coding, filing, revising, and compiling into books. The hospital-level system is the standardized management document of the hospital, and each volume is numbered. (3) Training at different levels to promote the implementation and improvement of the system: after the relevant system is formulated, the process of implementation is an extremely important link, and early effective and comprehensive implementation is an important prerequisite for ensuring its application effect. Before implementation, the system must be effectively trained. After the management system is compiled into a book, it will be distributed to relevant departments in a timely manner so that the staff of the hospital will be informed in time, and the staff will be trained at different levels. One is the training of medical personnel and administrative logistics personnel. Ordinary employees must make clear what the system is and be familiar with the various systems of the hospital. The second is the training of middle managers. This level of training must enable them to master the theory and practice of system management proficiently and to use various systems proficiently in their own work practice. The third is the training of hospital leaders. This level of training focuses on the theory and significance of the standardization of system management, how to improve the management efficiency of the hospital, and how to encourage leaders to give full attention and support. (4) The hospital has formulated the “Standardized Management System of Hospital Rules and Regulations” in the disposal stage and compiled “Hospital Rules and Regulations Compilation,” which is assigned to administrative management and clinical services. Various functional departments and clinical and medical technology departments have compiled and printed the Department Management Manual, Diagnosis and Treatment Guidelines and Operational Standards and compiled the Compilation of Key Processes of Patient Service and Hospital Management that matches the system.

### 2.3. Observation Indicator

#### 2.3.1. Evaluation of the Work Efficiency of Medical Staff

Before and after the study, the self-made work efficiency scale was adopted to quantify the work efficiency of medical staff. The scale has a total of 15 questions, and the full score for each item is 5 points. The higher the score, the higher the work efficiency.

#### 2.3.2. Evaluation of Teamwork among Medical Staff

Before and after the study, the self-made work efficiency scale was adopted to evaluate the team cooperation of medical staff [13]. The evaluation dimensions included team cohesion, team learning ability, team cooperation, team professional quality, and team spirit. The dimension contains a number of indicators, with a total score of 100 points. The higher the score, the better the team collaboration.

#### 2.3.3. Medical Staff Self-Identity Evaluation

Before and after the study, the self-identity scale included 19 items, which were scored on four levels from 1 to 4, and the total score of the questionnaire was calculated after the reverse items were scored in reverse. A higher score indicates that the subject's self-identity is well developed, and a lower score indicates that the subject's self-identity is still developing and forming [14].

#### 2.3.4. Management Quality Score Comparison

The management quality scores after the intervention were calculated.

#### 2.3.5. Comparison of the Incidence of Bad Behaviour

To observe the adverse events that occurred during the study.

### 2.4. Statistical Analysis

Using SPSS 21.0 statistical software, measurement data were examined for normal distribution and homogeneity of variance before statistical analysis to meet the requirements of a normal distribution or approximately normal distribution and were presented as $\bar{x}\pm s$. Repeated-measures data were analyzed by repeated-measures analysis of variance. The *t*-test was adopted for comparison between the two groups, and the count data were presented as *n*(%), and the chi-square test was adopted. *P* < 0.05 indicated that the difference exhibited statistically significant.

## 3. Results

### 3.1. Evaluation of the Work Efficiency of Medical Staff

**Figure 1:**
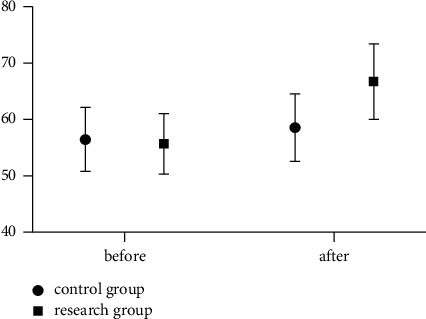
Comparison of work efficiency between the two groups.

Before the study, there exhibited no significant difference in the work efficiency scores of medical staff (*P* > 0.05); after the intervention, the work efficiency scores of the medical staff in the study group were significantly higher than that in the control group (*P* < 0.05). The specific results are indicated in Figure 1.

### 3.2. Evaluation of Teamwork among Medical Staff

**Figure 2:**
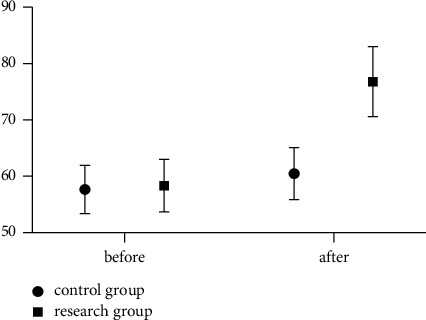
Comparison of team cooperation between the two groups of medical staff.

Before the study, there exhibited no significant difference in medical staff in the teamwork score (*P* > 0.05); after the intervention, the teamwork score of the medical staff in the study group was significantly higher than that in the control group (*P* < 0.05). The specific results are indicated in Figure 2.

### 3.3. Medical Staff Self-Identity Evaluation

Before the study, there exhibited no significant difference in medical staff's self-identity scores (*P* > 0.05); after the intervention, the self-identity score of medical staff in the study group was significantly higher than that in the control group (*P* < 0.05). The specific results are indicated in Table 2.

### 3.4. Management Quality Scores

After the intervention, the management quality score of the study group was significantly higher than that of the control group (*P* < 0.05). The specific results are indicated in Table 3.

### 3.5. Comparison of the Occurrence of the Bad Behaviour of Medical Staff

After the intervention, the incidence of medical staff in the study group was significantly lower than that in the control group (*P* < 0.05). The specific results are indicated in Table 4.

## 4. Discussion

Hospitals are one of the iconic windows of society [14]. To build the brand of a harmonious hospital and create a harmonious doctor-patient relationship, the hospital must pay attention to enhance its own quality management level, change the partial, fragmented, intermittent, and post-event control passive standard management model in the past, and focus on establishing an overall, continuous hospital quality management model with full participation [15]. (1) Current status of hospital quality management in my country: compared with large enterprises, the quality management of hospitals started relatively late, which was only non-systematic, non-modern quality management under the constraints of empirical management. Some work was carried out before the 1970s [16]. At present, hierarchical management is the main quality management mode in hospitals at all levels. Few hospitals can apply a systematic and enduring model of good quality management. Most of the senior managers of the hospital are not clear about the advanced quality management theory. Due to the particularity and professionalism of the medical industry, most hospital leaders are medical professionals, and they do not pay much attention to the establishment and promotion of quality management models [17, 18]. (2) Insufficient staffing and limited capacity of full-time quality management personnel: the effective operation of the hospital's quality management model depends on the strong implementation of full-time quality management personnel. Most hospitals have few full-time quality management personnel, and they do not come from quality management or related management majors. However, the characteristics of medical background overcome the obstacles of daily communication [19], but the lack of management expertise seriously affects its work efficiency and effectiveness. In addition, a considerable number of quality management personnel do not know enough about their work and feel that quality management work is dispensable in the hospital [20]. (3) Defects in quality assessment methods and unclear result orientation: my country has used rigid indicators such as cure rate, case fatality rate, and diagnostic coincidence rate for many years to measure the medical level and integrated them into some advanced quality management theories [21]. As a result, the evaluation results lack authenticity, cannot truly reflect the level of medical technology, and lack sensitivity and comparability. In addition, the result orientation of hospital quality management is not clear, resulting in the phenomenon of large false voids in the process of implementation, which cannot accurately reflect the quality level of the entire hospital [22]. (4) Insufficient investment in training and continuing education: good quality training is an effective guarantee for the operation of the quality management model. The survey found that many hospitals do not pay enough attention to quality training work. Usually, the training of relevant personnel is not comprehensive and in place, and they cannot timely continue to educate the full-time quality management personnel on advanced management theories, which makes the hospital's quality management level always stay at the general level, unable to move forward [23].

Hospital quality management is the process of using modern scientific management methods to manage and control the elements, processes, and results of medical services in accordance with the rules of hospital quality formation and the requirements of relevant laws and regulations to achieve system improvement and continuous improvement of hospital quality [24]. The quality of hospitals is directly related to the health rights and interests of the people and their personal experience of medical services. Continuously improving the quality of hospitals has become the core issue of the survival and development of hospitals. The use of quality management tools such as PDCA cycle to establish a hospital quality management model and continuous quality improvement through the cycle of PDCA has become one of the effective ways to enhance the core competition of hospitals [25]. In this study, 200 medical staff members from January 2020 to December 2021 in our hospital were prospectively enrolled as the research subjects, and 200 medical staff members were assigned to the control group and the research group using the numerical table method, with 100 cases in each group. The medical staff in the control group implemented the conventional system management method, while the research group comprehensively used PDCA and FMEA management tools on the basis of the implementation of the conventional system management to explore the effect of the comprehensive use of PDCA and FMEA management tools on the work efficiency, teamwork, and self-identity of medical staff. Influence: the results of the study found that before the study, there exhibited no significant difference in the work efficiency scores of medical staff; after the intervention, the work efficiency score of medical staff in the study group was higher than that in the control group; before the study, there exhibited no significant difference in the scores of teamwork of medical staff; after the intervention, the teamwork score of medical staff in the study group was higher than that in the control group; before the study, there exhibited no significant difference of medical staff's self-identity scores; after the intervention, the self-identity score of medical staff in the research group was higher than that in the control group; after the intervention, the management quality score in the research group was higher than that in the control group; after the intervention, the incidence of medical staff in the study group was remarkably lower than that in the control group.

FMEA is a bottom-up inductive system analysis or process analysis method adopted to assess potential errors, including finding out what could cause errors, the methods by which errors can occur (failure modes), and determining each failure The effect of the mode on the system [26]: FMEA is implemented according to 5 core steps: identifying themes, forming a multidisciplinary team, drawing a flowchart, conducting a hazard analysis, and developing and executing improvement actions. PDCA cycle management mode is mostly adopted for continuous quality improvement, and the effect is remarkable. FMEA is a potential hazard prediction technology that combines theory and experience, a systematic, feed-forward safety management analysis method, and a quality management tool; FEMA emphasizes pre-prevention rather than post-correction, and its purpose lies in the first line of defence blocking the source of defects, and its application helps to detect risks early in the process [27, 28]. The PDCA cycle was proposed by Dr. Deming, an American quality management expert in the 1950s. The main content is the abbreviation of plan, do, check, and action. It is the basis of total quality management. The PDCA cycle can be assigned to four stages, namely the planning stage, the implementation stage, the inspection stage, and the processing stage. These four stages can often be carried out successively or simultaneously. In actual work, the specific operations are as follows: work objectives are first formulated or questions are asked, then the problems are analyzed to find out the causes of the problems, and then corresponding measures are formulated and implemented [29–31]. During the implementation process, by evaluating the effect of implementation, the experience is summarized and transferred to the next cycle. It is an example of an effective management method of continuous improvement and spiral upward. The ultimate goal of management is to optimize various tasks [32–34]. The PDCA cycle model conforms to the objective law of “practice, cognition, practice, and cognition again and again” and reflects the scientific epistemology. Therefore, the PDCA cycle is considered to be a universal and practical management philosophy [35]. This standardized and scientific management procedure was first applied to enterprise management and achieved good results [36]. Due to its rigorous operating procedures and diverse management levels, the PDCA cycle is suitable for different types of management. Various industries adopt it as an effective method to strengthen internal management and is also suitable for various management work and management [37, 38]. The PDCA cycle management theory is applied to the hospital's internal system management, and it has significant management advantages, including the following: (1) PDCA cycle management theory stimulates the management subject's sense of responsibility by improving the staff and other management subjects' cognition of internal system management and work enthusiasm, to achieve the purpose of optimizing the internal management system of the hospital. (2) This management theory aims to maintain the normal operation of the hospital and improve the service quality and emphasizes the importance of each staff member [39]. (3) When the internal system manager loses absolute control and personal authority, it is to give each staff member the corresponding management power, fully support the work of each staff member, and enhance the harmonious communication between the staff members and through the management of the staff members. Professional guidance and training provide important prerequisites for staff to complete their work with high quality. (4) The work enthusiasm and sense of responsibility of the internal management staff are effectively stimulated, and the quality of internal management work is enhanced by understanding the work schedule and plan and clarifying the work goals. (5) The quality of the internal management work has been continuously improved, and the internal management work has been carried out smoothly with a plan and progress. The circular application of PDCA in the standardized management of the hospital management system enables employees to have rules to follow, clear goals, and clear responsibilities and rights in their work, enhances the work efficiency of each department, ensures work quality, and promotes system management satisfaction. During the implementation of the system, the weak points in the operation of the system are identified, the PDCA cycle and other quality control tools are used to rectify the weak points, and finally the hospital management system is enhanced. On the basis of supplementing and improving all aspects of the system, clarifying the internal relationship between systems and standardizing the internal management structure and power operation rules are focused so that the management systems of each part are closely connected like machine gears, management integration is formed, and the implementation of the system is strengthened [40]. In the process of implementing the system, the hospital still needs to start from itself, take the initiative to act, actively respond, and constantly explore and enhance the standardized management of the hospital management system; for existing problems, difficulties are overcome and problems are solved to ensure the real implementation of the system. Management is a dynamic decision-making process, and the system and hospital management should be effectively combined, using quality management tools combined with effective performance assessment, to promote the system in practice so that the hospital development can better meet the needs of the development of the times [41].

In summary, the application of PDCA and FMEA management tools in the internal management of the hospital can significantly improve the efficiency of the hospital's internal management, improve the work efficiency, teamwork, and self-identity of the medical staff, and contribute to a better development of the hospital. Limited by the sample size, the results of this study need to be confirmed by the enlarged central test.

## Figures and Tables

**Table 1:** Comparison of general data of the two groups of patients.

Group	*N*	Gender: (male/female)	Age (years)	Education level		
				Junior college	Undergraduate course	Graduate student
R group	100	58/42	35.53 ± 4.25	24	57	19
C group	100	54/46	34.53 ± 4.15	22	58	20
*χ* ^2^/*t*		0.324	1.683	0.121		
*P*		0.568	0.093	0.941		

**Table 2:** Comparison of self-identity of medical staff in two groups $\left(\bar{x}\pm s,d\right)$.

Group	*N*	Medical staff self-identity score		*t*	*P*
		Before intervention	After intervention		
C group	100	55.23 ± 4.26	55.64 ± 4.62	0.652	0.514
R group	100	56.22 ± 5.17	69.73 ± 5.13	18.549	0.000
*t*		1.477	20.409		
*P*		0.141	0.000		

**Table 3:** Comparison of management quality scores between the two groups $\left(\bar{x}\pm s,d\right)$.

Group	*N*	Management quality scores
C group	100	63.42 ± 5.23
R group	100	89.64 ± 6.82
*u/χ* ^2^		10.507
*P*		<0.01

**Table 4:** Comparison of the occurrence of adverse behaviours of medical staff in the two groups (*n*).

Group	*N*	Inefficiency	Insufficient business ability	Insufficient knowledge of management system
C group	100	24	12	32
R group	100	7	4	13
*χ* ^2^		11.032	4.347	10.351
*P*		<0.01	0.037	0.001

## Data Availability

The datasets used and analyzed during this study are available from the corresponding author upon reasonable request.
